# Neonatal hypocalcemia and hydrocephalus as early manifestations of intermediate osteopetrosis: successful hematopoietic stem cell transplantation despite negative genetic testing: a case report

**DOI:** 10.3389/fendo.2026.1910072

**Published:** 2026-07-30

**Authors:** Laura Figà, Daniele Franzone, Maura Faraci, Filomena Pierri, Sara Pestarino, Giulia Amico, Francesca Faravelli, Gianluca Piatelli, Anna Elsa Maria Allegri, Mohamad Maghnie, Flavia Napoli, Natascia di Iorgi

**Affiliations:** 1 Department of Neuroscience, Rehabilitation, Ophthalmology, Genetics, Maternal and Child Health (DINOGMI), University of Genoa, Genoa, Italy; 2 Pediatrics and Neonatology Unit of Savona, IRCCS Istituto Giannina Gaslini, Genoa, Italy; 3 Hematopoietic Stem Cell Transplantation Unit, IRCCS Istituto G. Gaslini, Genova, Italy; 4 Laboratory of Human Genetics, IRCCS Istituto Giannina Gaslini, Genoa, Italy; 5 Genomics and Clinical Genetics Unit, IRCCS Istituto Giannina Gaslini, Genova, Italy; 6 Neurosurgery Unit, IRCCS Istituto Giannina Gaslini, Genova, Italy; 7 Paediatric Endocrinology Unit, Department of Paediatrics, IRCCS Istituto Giannina Gaslini, Genoa, Italy

**Keywords:** case report, hematopoietic stem-cell transplantation, hydrocephalus, hypocalcaemia, osteopetrosis

## Abstract

Osteopetrosis is a rare genetic skeletal disorder caused by defective osteoclast-mediated bone resorption, leading to increased bone density and complications including hypocalcemia, pancytopenia, cranial nerve compression, and, more rarely, hydrocephalus. We report a rare case of intermediate osteopetrosis presenting with neonatal hypocalcemia and progressive macrocephaly. Brain magnetic resonance imaging confirmed obstructive hydrocephalus, which was treated with ventriculoperitoneal shunting. Clinical and radiological findings supported the diagnosis of osteopetrosis, although targeted genetic testing and whole exome sequencing did not identify a causative variant. The patient was treated with calcium gluconate and vitamin D supplementation, followed by hematopoietic stem cell transplantation from an HLA-matched unrelated donor using peripheral blood stem cells. This case highlights the importance of early recognition and timely hematopoietic stem cell transplantation to promote successful engraftment and improve long-term outcomes, even in the absence of molecular confirmation.

## 1 Introduction

Osteopetrosis is a rare genetic skeletal disorder characterized by defective osteoclast-mediated bone resorption, resulting in increased bone density, skeletal fragility, and a predisposition to fractures despite the apparent bone sclerosis (1–3). Additional clinical manifestations include alterations in calcium–phosphorus metabolism, craniosynostosis, hematologic abnormalities such as pancytopenia and immunodeficiency in severe forms, extramedullary hematopoiesis, and cranial nerve compression. According to inheritance pattern and clinical severity, osteopetrosis is classified into infantile malignant autosomal recessive, intermediate autosomal recessive, and late-onset autosomal dominant forms (4). Most autosomal recessive cases are associated with biallelic variants in the TCIRG1 gene. Autosomal dominant osteopetrosis typically presents during adolescence or adulthood, whereas autosomal recessive forms usually manifest in infancy with skeletal deformities, growth failure, hepatosplenomegaly secondary to bone marrow insufficiency, and cranial nerve compression leading to visual and hearing impairment (5). Neonatal hypocalcemia with tetany is an uncommon presentation, and hydrocephalus, usually secondary to aqueductal stenosis or impaired venous drainage caused by skull base sclerosis, represents a rare complication (6).

Only a limited number of cases of osteopetrosis associated with hydrocephalus have been reported in the literature, most of which were classified as severe autosomal recessive forms (**Table 1**) (7).

**Table 1:** Cases of osteopetrosis associated with hydrocephalus described in literature.

Author, Year	Study type	Patient characteristics		Genetics (gene)	Neonatal hypocalcemia	Sign/symptoms beyond hydrocephalus and low calcium	Treatment for hydrocephalus	Hematopoietic stem cell transplant
		Age (y)	Gender					
Al-Tamini Y., 2008	Case series	7	Female	AR	No	Developmental delay, multiple fractures, impaired vision	VPD	Not mentioned
		5	Female	AR	Yes	Nystagmus,	ETV	Not mentioned
		1,08	Male	AR	No	Nystagmus, difficulty in fixing gaze	VPD	Yes
Turgut M., 2010	Case report	2,2	Female	AR	No	Asymptomatic	VPD	Yes
Chate SV., 2011 (6)	Case report	0,5	Male	AR	No	Delayed development, poor growth, intermittent fever/abdominal distension, hepatosplenomegaly, anemia and thrombocytopenia, optic atrophy	Not mentioned	Candidate for HSCT
Essabar L., 2014	Case report	1,08	Male	AR (TCIRG1)	No	Hepatosplenomegaly	VPD	No
Scott W., 2014	Case series	0.75	Female	AR (TCIRG1)	Yes	Pancytopenia, splenomegaly	ETV	Not mentioned
Razik A., 2015	Case report	5	Male	AR	No	Difficulty walking/recurrent breathing difficulties, pancytopenia,	Lost to follow up	Lost to follow up
Stella I., 2017	Case report	0,58	Male	AR (TCIRG1)	Yes	Hepatosplenomegaly, bulging fontanella, anemia, thrombocytopenia.	Craniotomy	Yes
Isler C., 2018 (15)	Case report	7	Male	Negative (1^st^ level)	No	Tetraparesis, bilateral conductive hearing loss, papilledema, hepatosplenomegaly	VPD	Not mentioned
Lee A., 2021	Case report	0,5	Male	AR (TCIRG1)	No	Asyntomatic	VPD	Yes

AR, autosomal recessive; VPD, ventriculoperitoneal derivation; ETV, Endoscopic third ventriculostomy.

We describe an unusual case of intermediate osteopetrosis presenting with neonatal hypocalcemia and obstructive hydrocephalus.

## 2 Case description (patient information and clinical findings)

An 11-month-old boy was referred to our Pediatric Endocrinology Unit following a recent fracture of the right forearm. The patient was an adopted child with a history of early abandonment; therefore, prenatal, perinatal, and family history information was largely unavailable. He was born full term, with birth weight and length appropriate for gestational age according to Italian neonatal anthropometric charts (weight: +1.26 SDS; length: +0.1 SDS). Head circumference at birth was 37 cm (>97th percentile; +2.38 SDS), consistent with macrocephaly.

On the fifth day of life, the patient developed symptomatic hypocalcemia characterized by limb muscle spasms. Serum total calcium was markedly reduced at 4.7 mg/dL (reference range 8.1–10.4 mg/dL [2.0–2.6 mmol/L]). Oral calcium gluconate (13.5 mg Ca++/kg/day) and cholecalciferol (vitamin D3, 400 IU/day) were initiated, and the patient was discharged on the ninth day of life.

At 24 days of age, he was readmitted because of persistent symptomatic hypocalcemia. Cranial ultrasonography excluded cerebral hemorrhage and major brain malformations. Laboratory investigations showed persistent hypocalcemia (7.2 mg/dL; reference range 8.1–10.4 mg/dL [2.0–2.6 mmol/L]), hyperphosphatemia (7.2 mg/dL; reference range 2.6–4.5 mg/dL [0.84–1.45 mmol/L]), elevated parathyroid hormone levels (210.9 pg/mL; reference range 12–98 pg/mL), increased alkaline phosphatase (317 U/L; reference range 30–120 U/L), and elevated calcitonin levels (19.8 pg/mL; reference range 0–10 pg/mL). Blood cultures, urinalysis, and 24-hour urinary electrolyte evaluation were unremarkable. Thyroid and parathyroid ultrasonography showed physiological thymic hyperplasia. Conventional karyotyping revealed a normal 46,XY male karyotype, while comparative genomic hybridization (CGH) array identified a 22q11.23 microduplication classified as a variant of uncertain significance.

Oral calcium gluconate and vitamin D3 supplementation were progressively increased up to 45 mg Ca++/kg/day and 800 IU/day, respectively, resulting in normalization of serum calcium levels.

At two months of age, weight was 4.9 kg (36th percentile), length 58 cm (55th percentile), and head circumference 42.5 cm (>99th percentile). Due to the rapid increase in head circumference, cranial ultrasonography was repeated and revealed bilateral asymmetric ventriculomegaly (more pronounced on the left side) associated with a dysmorphic right lateral ventricle.

The patient was urgently referred to the neurosurgery department. Brain magnetic resonance imaging (MRI) demonstrated mild enlargement of the ventricular system and subarachnoid spaces. Associated cranial abnormalities included extensive craniolacunar changes at the vertex, abnormal morphology of the anterior cranial fossa with “hook-like” prominence of the anterior clinoid processes compressing the olfactory tracts, and mild stenosis of the optic foramina with early bilateral optic nerve sheath prominence. Brain computed tomography (CT) confirmed craniolacunia, showing marked widening of the bregmatic fontanelle and of the coronal, sagittal, and metopic sutures, resulting in posterior trigonocephaly.

**Figure 1:**
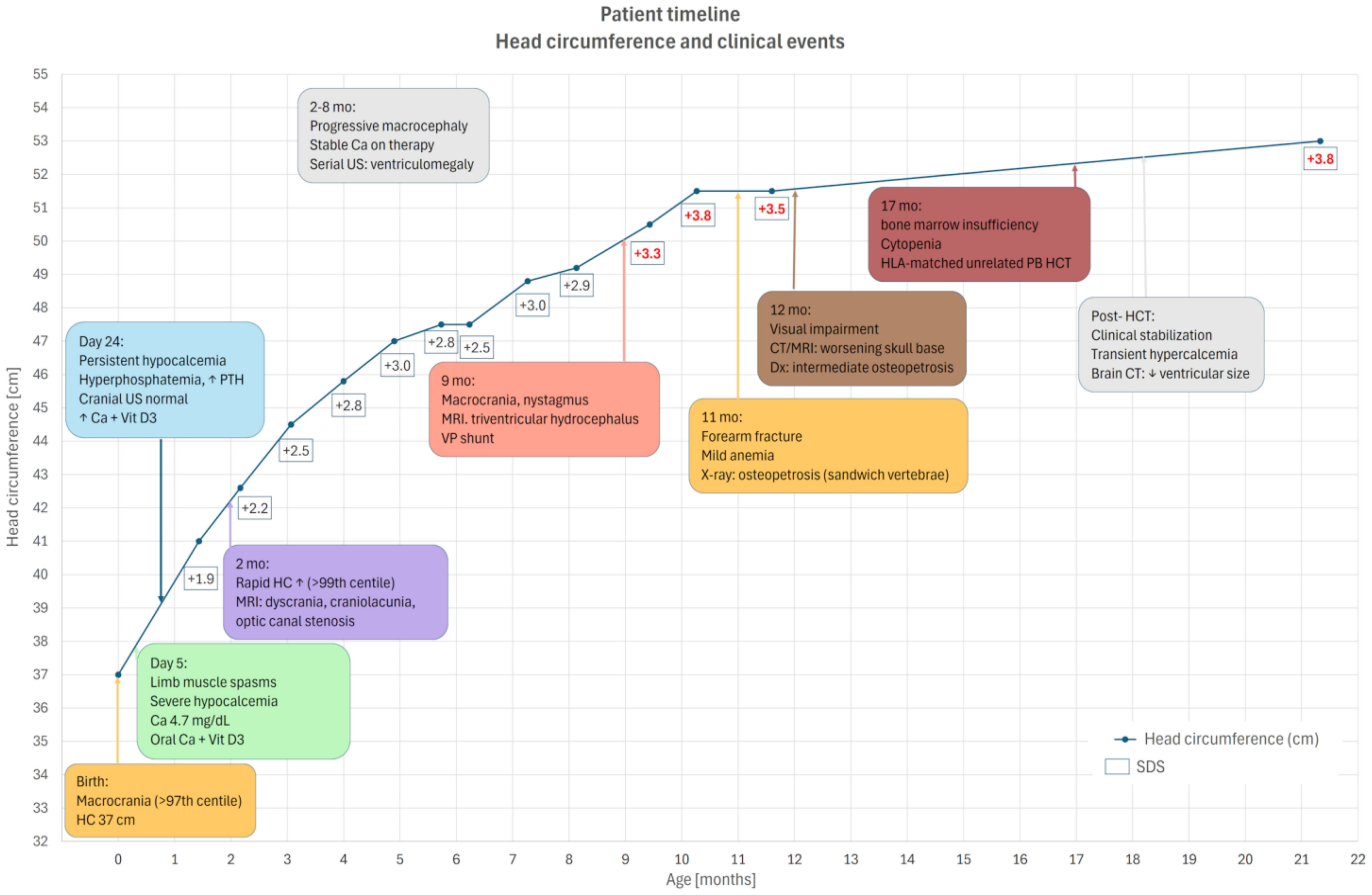
Head circumference (HC) trajectory of the patient from birth to 11 months and 18 days with corresponding SDS. Colored boxes highlight key clinical events during the patient's course. HC, head circumference; VP shunt, ventriculoperitoneal shunt; PB HCT, peripheral blood hematopoietic cell transplantation; Dx, diagnosis.

Ophthalmologic evaluation was unremarkable, and the patient was discharged with planned clinical and ultrasonographic follow-up. Over the following months, progressive macrocephaly and worsening ventricular enlargement were observed (**Figure 1**). At nine months of age, repeat neurosurgical assessment was performed. Brain CT demonstrated progression of the dyscranial abnormalities, while MRI confirmed triventricular hydrocephalus. Ventriculoperitoneal shunting was therefore successfully performed.

From a neurodevelopmental perspective, at nine months of age the patient exhibited delayed developmental milestones. Head control remained poor during pull-to-sit maneuver, independent sitting had not yet been achieved, and generalized hypotonia with reduced muscle strength was present, more pronounced on the left side.

During hospitalization, recurrent hypocalcemia was detected, likely related to progressive tapering of oral calcium supplementation during the previous months. Calcium gluconate therapy was therefore restarted at 4.5 mg Ca++/kg/day together with vitamin D3 at 800 IU/day.

Despite the presence of hydrocephalus, anterior pituitary function remained preserved, with cortisol, FT4, and IGF-1 levels within the normal range (**Table 2**).

**Table 2:** Patient’s blood tests at initial assessment in endocrinology unit.

Variable	Value	Normal range
Hemoglobin	**9,2 g/dl**	11-13
Red blood cells	3,49 x 10^6/μL	3,6-5
White blood cells	13,85 x 10^3/μL	13,8-15,3
Platelets	550x10^3/μL	150-450
Calcium	4,53 mEq/L	4,05-5,2
Ionized calcium	**1,13 mmol/L**	1,15-1,29
Parathyroid hormone	**99 pg/ml**	15-65
Vitamin D	33,11 ng/ml	20-100
1-25OH Vitamin D	352,6 pmol/L	36,5-316,2
Cortisol	17,7 µg/dl (488.3 nmol/L)	2,5-19,5 µg/dl (69-537.9 nmol/L)
FT4	1,41 ng/dl (18.1 pmol/L)	1,04-1,59 ng/dl (13.4-20.5 pmol/L)
TSH	1,36 µU/ml (1.36 IU/L)	1-6.7 µU/ml (1-6.7 IU/L)
IGF-1	16,4 ng/ml (2.14 nmol/L)	11.8-96.4 ng/ml (1.54-12.6 nmol/L)

Altered values are represented in bold font. The Units of measurement of the International System are given in parenthesis. FT4, free thyroxine; TSH, thyrotropin (thyroid-stimulating hormone); IGF-1, insulin-like growth factor-1.

## 3 Timeline

### 3.1 Diagnostic assessment, therapeutic Intervention, follow up and outcome

**Figure 2:**
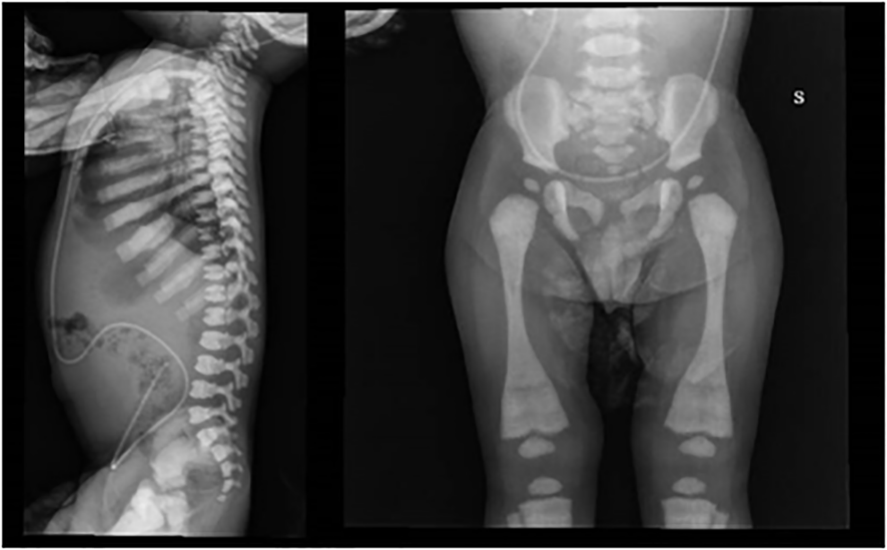
**(A)** sclerosis of vertebral endplate: "sandwich vertebrae"; **(B)** increased bone density of vertebrae and peripheral area of iliac wings, ischiopubic rami, and acetabular roof.

Intermediate osteopetrosis was diagnosed based on clinical and radiological findings (**Figure 2**). Given the reduced size of the optic canals observed on brain MRI and the presence of nystagmus, further investigations were performed to assess possible involvement of the second and eighth cranial nerves. Audiological evaluation was unremarkable, whereas visual evoked potentials demonstrated a mild increase in optic nerve conduction latency. Urinalysis, 24-hour urinary electrolyte assessment, and urinary pH were within normal ranges, thereby excluding renal tubular acidosis. Abdominal ultrasonography showed no evidence of nephrocalcinosis or hepatosplenomegaly.

A fast-track NGS *in silico* panel, obtained through whole exome sequencing (WES) (Whole Exome WES_v2 - Sophia Genetics, Nextseq Sequencing Platoform - Illumina), including genes associated to osteopetrosis (8) (ANKH, CA2, CLCN7, CSF1R, CTSK, FAM20C, FERMT3, IKBKG, LEMD3, LRP5, LRRK1, OSTM1, PLEKHM1, PTH1R, RASGRP2, SLC4A2, SLC29A3, SNX10, TCIRG1, TGFB1, TNFSF11, TNFRSF11A, TYROBP) was performed and tested negative. A secondary analysis of the 5’-UTR portion (9) and intron 15 mutational hotspot (10) in the TCIRG1 gene was carried out via Sanger Sequencing and resulted negative. Due to initial ocular and hematological involvement, this patient was a candidate for HCT as the genetic analysis had not revealed causative variants contraindicating the procedure (e.g. in *OSTM1 or TNFSF11*) (4).

After optimization of oral calcium gluconate therapy (13 mg Ca++/kg/day) and vitamin D3 supplementation (800 IU/day; 125 IU/kg/day), the patient was discharged and referred to the Hematopoietic Transplant Unit. At 17 months of age, he underwent peripheral blood stem cell transplantation (PBSC) from an HLA-matched unrelated donor (MUD). He continued oral calcium supplementation at the same dose (13mg/Kg/day) until conditioning, then switched to parenteral nutrition, which contained the recommended daily calcium requirements for age and weight. A myeloablative conditioning regimen was administered, consisting of thiotepa 8 mg/kg (day −7), treosulfan 10 mg/m²/day (days −6 to −4), and fludarabine 40 mg/m²/day (days −6 to −3).

Graft-versus-host disease (GVHD) prophylaxis included cyclosporine (Csa) (initially 1 mg/kg/day, increased to 3 mg/kg/day from day −1), mycophenolate mofetil (30 mg/kg/day), and anti-T-lymphocyte globulin (ATLG Grafalon^®^), 10 mg/Kg/day for 3 days before HCT). Csa levels between 150–200 ng/mL were maintained to prevent rejection and acute GVHD). Defibrotide (25 mg/kg/day) was administered for prevention of hepatic veno-occlusive disease, while acyclovir (30 mg/kg/day from day 0) was used for herpesvirus prophylaxis. Because Epstein–Barr virus (EBV) positivity was detected before transplantation (9750 copies in whole blood and 151 copies in lymphomonocytes), rituximab (200 mg/m²) was administered on day −1 as prophylaxis against post-transplant lymphoproliferative disorder (PTLD).

Neutrophil engraftment, defined as the first of three consecutive days with an absolute neutrophil count >0.5 × 10^9^/L, was achieved on day +19. Platelet engraftment, defined as a platelet count >50 × 10^9^/L maintained for at least 7 days without transfusion support, was achieved on day +11. Chimerism analysis performed on day +170 demonstrated complete donor engraftment (100% donor cells).

On day +7 post-transplant, the patient developed transient hypercalcemia, with ionized calcium levels rising to 1.78 mmol/L (reference range 1.15–1.29 mmol/L) and total calcium to 3.40 mmol/L (reference range 2.03–2.60 mmol/L). This complication was successfully managed with intravenous hydration, furosemide, methylprednisolone (1 mg/kg), and intravenous clodronate (150 mg/day for 5 days), resulting in normalization of calcium levels. Due to recurrent hypercalcemia two weeks later, an additional short course of clodronate was administered (150 mg on alternate days for 6 days).

On day +39, the patient developed grade 2 acute intestinal GVHD, which responded well to steroid therapy. No additional acute or chronic transplant-related complications were observed.

Four months after HSCT, brain CT demonstrated reduction in ventricular size, suggesting improvement of the hydrocephalus, together with mild improvement in skull base foraminal stenosis, although cranial vault thickening persisted. Dental evaluation performed eight months post-transplant showed delayed dentition and persistence of dental buds, findings considered consistent with the underlying disease.

At 19 months after HSCT, weight was 11.6 kg (slightly below the 3rd percentile) and height was 87.5 cm (between the 3rd and 10th percentiles). Neurodevelopmental evaluation revealed persistent global developmental delay. Independent walking was achieved at 2 years and 6 months of age, although gait instability remained present, and expressive language was limited to single words or short word combinations. Skeletal radiography performed at the same time demonstrated resolution of bone sclerosis.

At the most recent follow-up (19 months post-HSCT), hematopoietic function had normalized, with white blood cells 6.8 × 10^9^/L (reference range 4.3–11.9 × 10^9^/L), hemoglobin 126 g/L (reference range 120–140 g/L), and platelet count 320 × 10^9^/L (reference range 150–400 × 10^9^/L).

## 4 Discussion

We report a case of intermediate osteopetrosis presenting with neonatal hypocalcemia, progressive macrocephaly secondary to obstructive hydrocephalus, anemia, and early ocular involvement, successfully treated with hematopoietic stem cell transplantation (HCT) from an unrelated donor. Assessment of inheritance was limited because the patient had been adopted and no biological family history was available. Nevertheless, the clinical presentation was more consistent with an autosomal recessive form, whereas autosomal dominant osteopetrosis was considered unlikely given its typically milder phenotype and later onset (4).

Despite the early presentation with profound neonatal hypocalcemia, the overall phenotype and clinical course were considered more compatible with intermediate osteopetrosis than with classic malignant osteopetrosis, given the absence of hepatosplenomegaly and of rapidly progressive, severe, hematological impairment during early infancy, despite the presence of early ocular involvement.

A PubMed search using the terms “osteopetrosis” and “hydrocephalus” identified 12 case reports and 2 case series, corresponding to a total of 19 patients. Among these, only three cases described the association of osteopetrosis with both hydrocephalus and hypocalcemia. Eight patients (five isolated case reports and three cases from a four-patient series) were excluded from comparison because genetic data were unavailable. Almost all reported patients had autosomal recessive osteopetrosis; when specified, the causative gene was TCIRG1, encoding the osteoclast-specific vacuolar proton pump (V-ATPase α3 subunit), which is essential for acidification of the resorption lacuna, vesicle trafficking, and ruffled border formation (3, 4, 11).

More than half of patients with autosomal recessive infantile osteopetrosis harbor pathogenic variants in TCIRG1, whereas CLCN7 variants account for approximately 13% of cases (12). Additional genes associated with osteopetrosis include SNX10, OSTM1, TNFRSF11A, TNFSF11, and PLEKHM1 (13). Pathogenic variants in these genes impair osteoclast-mediated bone resorption (3). Nevertheless, a subset of patients remains without molecular confirmation despite extensive genetic testing (14). For example, Isler et al. (15) reported a 7-year-old patient with osteopetrosis, hydrocephalus, hepatosplenomegaly, and neurological impairment in whom genetic investigations were unremarkable.

In our patient, analysis of an osteopetrosis-associated virtual gene panel derived from next-generation sequencing (NGS) data, including 23 known disease-associated genes (8), did not identify pathogenic variants. Although NGS-based approaches represent standard diagnostic tools for osteopetrosis (16), they may fail to detect deep intronic or non-coding variants located outside routinely analyzed regions (14). Additional targeted analyses of the TCIRG1 gene, including the 5′-UTR region and the intron 15 mutational hotspot, also yielded negative results. Therefore, although a molecular diagnosis could not be established, the clinical and radiological presentation strongly supported the diagnosis of osteopetrosis.

After exclusion of genetic forms considered contraindications to transplantation, such as OSTM1- or TNFSF11-related disease, and in the absence of evidence of neurodegenerative involvement, HCT was performed despite the lack of molecular confirmation because of progressive bone marrow dysfunction and optic nerve compression, both recognized indications for urgent treatment.

Previous studies have shown improved survival and engraftment rates when HCT is performed early in life, particularly before 10 months of age (17). Timing of transplantation therefore represents a critical prognostic factor (3). Donor compatibility also significantly influences long-term outcomes, with reported 5-year overall survival rates after HLA-matched unrelated donor HCT ranging from 42% to 46% in malignant infantile osteopetrosis (18, 19).

Engraftment failure remains one of the most frequent post-transplant complications, especially in children transplanted after 10 months of age. In these cases, T-cell–replete haploidentical transplantation with post-transplant cyclophosphamide has been proposed as an alternative strategy (20). In our patient, complete donor chimerism (100%) was achieved by day +170, with neutrophil engraftment on day +19 and platelet engraftment on day +11 (18).

Post-transplant complications included grade 2 acute intestinal graft-versus-host disease (GVHD), which responded well to treatment with corticosteroids, cyclosporine, and mycophenolate mofetil, as well as transient hypercalcemia managed with hydration, diuretics, corticosteroids, and bisphosphonates. Hypercalcemia is a recognized early post-transplant event related to restoration of donor-derived osteoclast activity and may represent an indirect marker of successful engraftment (18, 21, 22).

No hepatic toxicity or veno-occlusive disease occurred, despite the relatively high incidence of these complications in osteopetrosis patients undergoing HSCT (19). Importantly, no major neurological, respiratory, or renal complications were observed during follow-up (18).

Our patient achieved complete engraftment without major chronic complications at 18 months after HSCT. Although longer follow-up is required, the clinical outcome appears encouraging. Emerging therapeutic strategies for osteopetrosis include *in utero* HSCT, gene therapy, and RANK/RANKL-targeted approaches (3, 23).

This case highlights the importance of early recognition and timely transplantation in osteopetrosis, even in the absence of molecular confirmation. When clinically feasible, whole genome sequencing should be considered to improve diagnostic sensitivity. Moreover, neonatal hypocalcemia associated with progressive macrocephaly or hydrocephalus should be recognized as a potential early red flag for osteopetrosis, even though these manifestations are infrequently reported (19).

## 5 Patient perspective

“Prior to HCT, which was performed when he was 1 year 5 months old, our son was not able to either hold his head up steadily or crawl. He started crawling a few months after the procedure, with independent ambulation achieved 1 year and 4 months following HCT. He has since progressed to stairclimbing and running. Although he has experienced occasional falls, no further bone fractures have occurred. His head had an uneven shape before HCT, while now its shape looks more round and regular. His bone pain has improved substantially: before HCT, our son would cry even during diaper change, while movement does not seem to cause him any pain any longer. He is still undergoing rehabilitation – both motor and visual – with constant improvement”.

## Data Availability

The original contributions presented in the study are included in the article/supplementary material. Further inquiries can be directed to the corresponding authors.
